# Diabetic Retinopathy Grading and Concurrent Cardiorenal Biomarker Abnormalities in Type 2 Diabetes: Albuminuria and Elevated NT-proBNP—A Retrospective Cross-Sectional Study

**DOI:** 10.3390/jcm15156106

**Published:** 2026-08-06

**Authors:** İrfan Alisan, Ahmet Gazi Mustan, Bektas Isik, Fatih Necip Arıcı, Cahit Dinçer, Çisem Yılmaz, Mehmet Erdevir, Ahmet Altıntaş, Çiğdem Erhan, Merve Saracoglu Sumbul, Huseyin Ali Ozturk, Erdinc Gülümsek, Begüm Seyda Avcı, Hilmi Erdem Sumbul

**Affiliations:** 1 Department of Internal Medicine, University of Health Sciences, Adana Health Practice and Research Center, Adana 01230, Türkiye; agazi_mstn@hotmail.com (A.G.M.); beko44485@gmail.com (B.I.); fatihneciparici803@gmail.com (F.N.A.); cahitdincer1164@hotmail.com (C.D.); cisemkizildag.33@gmail.com (Ç.Y.); drozturkhuseyinali@gmail.com (H.A.O.); drerd84@yahoo.com.tr (E.G.); begumtngnr@hotmail.com (B.S.A.); erdemsumbul@gmail.com (H.E.S.); 2 Department of Internal Medicine, Yuregir State Hospital, Adana 01240, Türkiye; erdevirmehmet@gmail.com; 3 Department of Internal Medicine, University of Health Sciences, Osmaniye Health Practice and Research Center, Osmaniye 80010, Türkiye; drahmetaltintas1@gmail.com; 4 Department of Internal Medicine, Seyhan State Hospital, Adana 01150, Türkiye; drcerhan@gmail.com; 5 Department of Public Health, Adana City Research and Training Hospital, Adana 01230, Türkiye; mervessumbul@gmail.com

**Keywords:** diabetic retinopathy, NT-proBNP, UACR, nephropathy, NT-proBNP elevation, microvascular complications, type 2 diabetes, cardiorenal association

## Abstract

**Background:** Diabetic retinopathy (DR) is the most prevalent microvascular complication of type 2 diabetes mellitus (T2DM) and a recognised marker of systemic vascular injury. Whether DR—graded independently by two experienced ophthalmologists as part of routine institutional care—is independently associated with concurrent nephropathy (urine albumin-to-creatinine ratio [UACR] ≥ 30 mg/g) and subclinical cardiac stress (N-terminal pro-B-type natriuretic peptide [NT-proBNP] ≥ 125 pg/mL) remains insufficiently examined. **Methods:** Retrospective cross-sectional study of 401 T2DM adults who underwent dilated fundus examination independently graded by two board-certified ophthalmologists (each ≥ 10 years’ experience in diabetic eye disease) using ETDRS-based classification. Inter-physician agreement was quantified by Cohen’s weighted kappa. The primary composite outcome was concurrent nephropathy and subclinical cardiac stress. Multivariable logistic regression adjusted for age, sex, HbA1c, diabetes duration, eGFR, hypertension, and pharmacological treatments. **Results:** The composite outcome was present in 120 (64.2%) DR-positive vs. 14 (6.5%) DR-negative patients (crude OR: 25.59 (13.78–47.51); *p* < 0.001; fully adjusted OR: 21.49 (11.02–41.81); *p* < 0.001). Inter-physician agreement was high (Cohen’s weighted κ = 0.88; 95% CI: 0.83–0.93). Nephropathy alone was found in 164 (87.7%) vs. 76 (35.5%) patients (OR: 12.95 (7.71–21.75); *p* < 0.001), and elevated NT-proBNP in 137 (73.3%) vs. 39 (18.2%) patients (OR: 12.29 (7.65–19.76); *p* < 0.001). A significant trend across DR severity grades was observed (*p* for trend < 0.001), although the gradient was not strictly monotonic. ROC AUC = 0.837 (95% CI: 0.784–0.860). **Conclusions:** Ophthalmologist-graded DR was independently associated with the concurrent presence of albuminuria and elevated NT-proBNP in T2DM. Because the design is cross-sectional, these findings describe association rather than prediction or causation. They generate the hypothesis that retinal grading may help flag patients in whom cardiorenal biomarker assessment is worth considering, but prospective validation is required before any screening application.

## 1. Introduction

Diabetes mellitus (DM) represents one of the most consequential public health challenges of the 21st century, with global prevalence exceeding 537 million adults and projected to surpass 780 million by 2045 [1]. T2DM drives a spectrum of microvascular complications—including diabetic retinopathy (DR), nephropathy, and neuropathy—alongside macrovascular disease affecting the coronary, cerebrovascular, and peripheral arterial systems. [1,2] Adults with T2DM face a two- to four-fold higher cardiovascular risk than normoglycaemic counterparts, and younger age at diagnosis is independently associated with progressively greater risk of both micro- and macrovascular disease [2].

Diabetic retinopathy is the most prevalent microvascular complication of DM, affecting approximately one-third of all diabetic individuals globally and constituting the leading cause of preventable vision loss among working-age adults [3]. Regular fundoscopic evaluation by trained ophthalmologists remains the clinical reference standard for DR detection and severity grading, providing high-resolution direct visualisation of retinal vascular pathology including microaneurysms, intraretinal haemorrhages, neovascularization, and macular oedema [3,4]. The Early Treatment Diabetic Retinopathy Study (ETDRS) severity scale offers a validated, reproducible five-category classification framework from no DR to proliferative DR (PDR) [4,5]. When performed by experienced ophthalmologists, fundus examination yields high inter-rater agreement, with published kappa values consistently exceeding 0.80 in expert panel settings [4,5].

Beyond its ophthalmological role, the retinal vasculature is increasingly recognised as a non-invasive window to systemic vascular health in diabetes [3,6]. Retinal and glomerular microvasculature share critical architectural homologies: both are composed of fenestrated capillaries with pericyte coverage and basement membranes selectively vulnerable to hyperglycemia-driven endothelial injury, oxidative stress, and advanced glycation end-product accumulation [6,7,8,9].

N-terminal pro-B-type natriuretic peptide (NT-proBNP) is a guideline-endorsed biomarker of myocardial wall stress and subclinical ventricular dysfunction, frequently elevated in T2DM patients even in the absence of symptomatic heart failure, reflecting early diabetic cardiomyopathy [10,11]. A landmark hospital-based cohort demonstrated that NT-proBNP in the highest tertile was independently associated with an OR of 13.78 for diabetic retinopathy and 6.23 for nephropathy, positioning it as a global biomarker of diabetic organ damage [11]. NT-proBNP has also been shown to correlate with markers of subclinical atherosclerosis—including pulse wave velocity and ankle-brachial index—in T2DM patients [12]. A prospective European case–cohort study further confirmed that higher NT-proBNP is independently associated with both microvascular (HR: 1.20) and macrovascular (HR: 1.37) complications among individuals who develop T2DM [13].

The urine albumin-to-creatinine ratio (UACR) is the guideline-recommended marker of diabetic nephropathy, reflecting glomerular endothelial damage and predicting progression to chronic kidney disease (CKD) and end-stage renal disease [14]. Evidence from the Adolescent Cardio-Renal Intervention Trial (AdDIT) demonstrated that even within the normoalbuminuric range, higher UACR tertiles independently predict retinopathy progression (HR: 2.1; 95% CI: 1.3–3.3), underscoring the bidirectional retinal–renal relationship [15].

Despite the biological coherence of a retinal–renal–cardiac axis in T2DM, few studies have systematically quantified the co-occurrence of nephropathy (elevated UACR) and subclinical cardiac stress (elevated NT-proBNP) in patients stratified by DR status confirmed through a protocol of independent grading by two experienced ophthalmologists with documented inter-physician agreement. The association between DR and nephropathy is itself well established; the less-well-characterised question, and the one addressed here, is how albuminuria and NT-proBNP behave together in relation to ophthalmologist-graded retinopathy. If ophthalmologist-confirmed DR proves to be independently associated with simultaneous cardiorenal biomarker abnormalities, this would support further prospective study of whether routine fundus examination—already embedded within standard diabetic care guidelines [16]—carries information relevant to cardiorenal evaluation.

The primary objective of this retrospective cross-sectional study is to determine whether DR graded by two independent experienced ophthalmologists is independently associated with a significantly higher prevalence of concurrent nephropathy (UACR ≥ 30 mg/g) and subclinical cardiac stress (NT-proBNP ≥ 125 pg/mL) in patients with T2DM. Secondary objectives included quantification of inter-physician reproducibility (Cohen’s kappa), analysis of individual biomarker associations, analysis of the gradient across DR severity grades, and ROC-based assessment of the discriminative performance of the full model within this sample.

## 2. Material and Method

### 2.1. Study Design and Setting

This was a retrospective cross-sectional study conducted at Adana City Training and Research Hospital. Electronic health record (EHR) data were extracted for the period [January/2025–December/2025]. No study-specific procedure, examination, or laboratory test was performed for this analysis: every fundus examination, UACR measurement and NT-proBNP measurement reported here had already been carried out and documented as part of routine clinical care before the study was conceived. The study was conducted in accordance with the Declaration of Helsinki and was approved by the Institutional Review Board (Approval date: 30 April 2026; Protocol No:25-1326). This approval date postdates the close of the entire data window (December 2025) by more than four months, which establishes that no assessment analysed here could have been performed for research purposes. The requirement for individual informed consent was waived by the Institutional Review Board given the retrospective, non-interventional design and the exclusive use of de-identified, routinely collected data. Reporting follows the Strengthening the Reporting of Observational Studies in Epidemiology (STROBE) checklist [17].

### 2.2. Participant Selection

**Figure 1:**
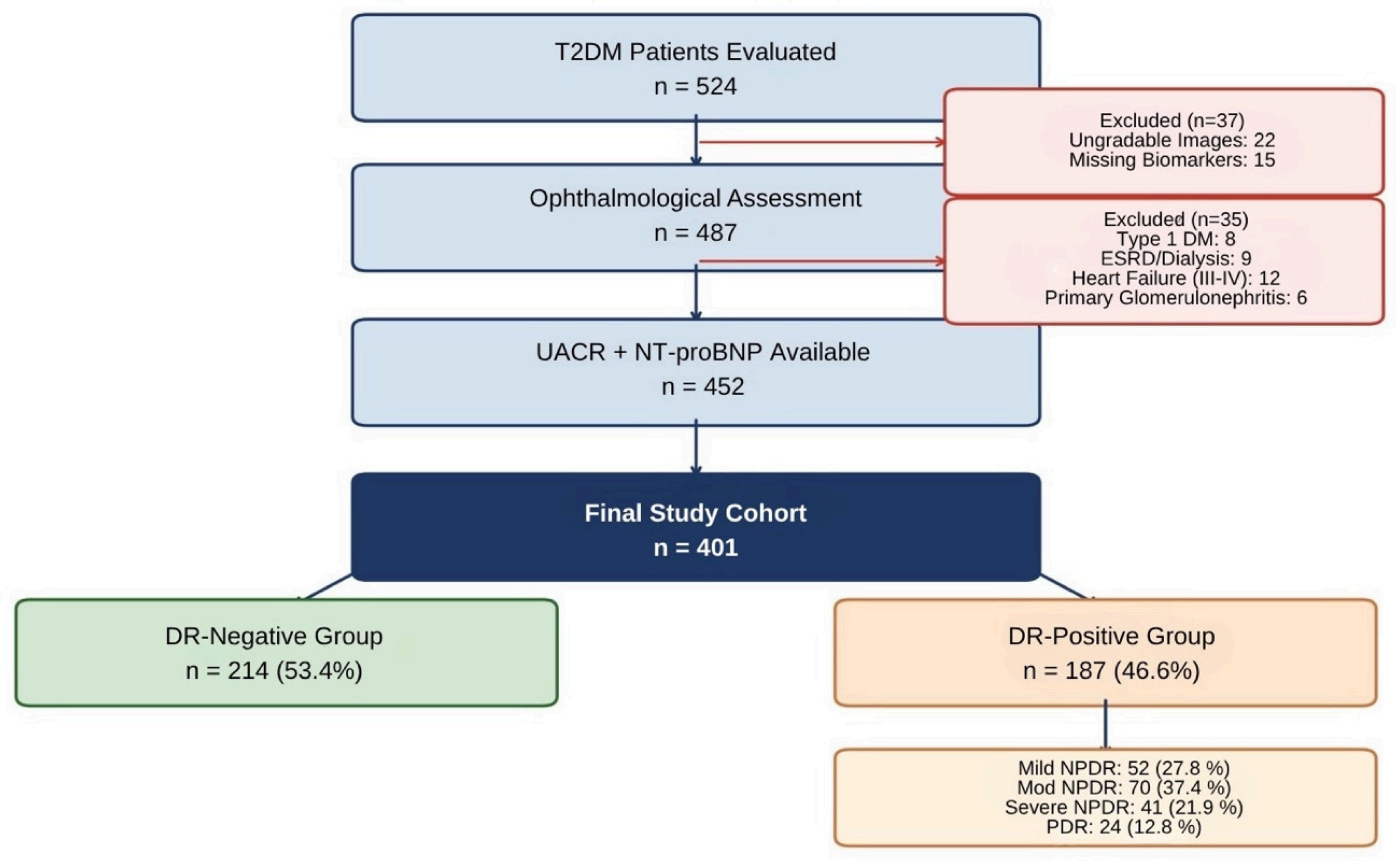
Study flow Diagram (STROBE).

Eligible participants were adults (≥18 years) with confirmed T2DM (ICD-10: E11.x) who underwent dilated fundus examination by both study ophthalmologists within the study period and had UACR and NT-proBNP measurements available within a ±90-day window of retinal assessment. Exclusion criteria: (1) type 1 diabetes mellitus; (2) end-stage renal disease (eGFR < 15 mL/min/1.73 m^2^) or current renal replacement therapy; (3) established heart failure NYHA class III–IV; (4) non-diabetic primary nephropathy; (5) media opacity precluding adequate fundus visualisation; and (6) pregnancy and atrial fibrillation. Patient selection is illustrated in Figure 1.

### 2.3. Ophthalmological Assessment Protocol

All patients underwent complete ophthalmic examination including best-corrected visual acuity, intraocular pressure measurement, and standardised slit-lamp biomicroscopy, followed by binocular indirect ophthalmoscopy and slit-lamp fundus examination with a 90-diopter condensing lens after pharmacological pupil dilation with tropicamide 1% and phenylephrine 2.5%. Fundus photography (45°, macula- and disc-centred) was performed to support documentation [3,4].

At our institution, dilated fundus examination of patients with diabetes is documented by two board-certified ophthalmologists working within the same diabetic eye service, each with a minimum of 10 years of dedicated clinical experience in diabetic eye disease. This dual-documentation workflow is an internal institutional practice rather than a requirement of any national Turkish guideline; the Turkish Ministry of Health and Turkish Ophthalmological Association recommendations, like the ADA Standards of Care [16], specify the frequency and modality of screening but do not mandate two graders. Because the workflow was already in place, the study cohort was assembled by retrospectively selecting those patients for whom two independent gradings were already present in the record; patients graded by a single ophthalmologist during the study period were not eligible. Each ophthalmologist entered a grade into the electronic record without access to the other’s entry, and both graded in the course of clinical care without knowledge of the present research question, which was formulated only after the data window had closed. The ETDRS-based five-category grading scale was applied: Grade 0 (No DR), Grade 1 (Mild NPDR), Grade 2 (Moderate NPDR), Grade 3 (Severe NPDR), and Grade 4 (PDR) [3,4,5].

In cases of discordance between the two recorded grades at the category level, the two ophthalmologists jointly reviewed the archived fundus photographs and examination notes during data abstraction and assigned a consensus grade through structured discussion; this adjudication step was performed for the purposes of the present analysis and was applied uniformly according to a procedure agreed before any biomarker data were linked to the retinal grades. The consensus grade was used as the definitive DR classification for all analyses. Patients were dichotomized as DR-positive (grade ≥ 1) or DR-negative (grade 0) for the primary analysis. Inter-physician agreement was quantified using Cohen’s weighted kappa (κ) with quadratic weighting, interpreted per the Landis-Koch scale.

### 2.4. Laboratory Assessment and Temporal Alignment

UACR was measured on a spot (untimed) urine sample using an immunoturbidimetric albumin assay with creatinine determined by the enzymatic method. NT-proBNP was measured by with manufacturer-stated (Roche, Mannheim, Baden-Württemberg, Germany) measuring. eGFR was calculated using the CKD-EPI 2009 creatinine equation. All samples were drawn in the outpatient setting during routine follow-up under standard, non-fasting-independent conditions; no sample was drawn in an emergency or inpatient context.

NT-proBNP is not a guideline-mandated test in the routine follow-up of uncomplicated type 2 diabetes. In our institution, it is obtained selectively, most often when a clinician is assessing dyspnoea, oedema, poorly controlled hypertension, or cardiorenal risk in a patient with long-standing diabetes. Every NT-proBNP value analysed here was ordered by the treating clinician for such an indication before the study existed, and none was ordered for research purposes. We now state explicitly that this introduces indication-based selection: patients with an available NT-proBNP are, by construction, patients in whom a clinician already suspected cardiorenal involvement, and the prevalence figures reported here should not be read as population prevalence estimates. This limitation is discussed in Section 4.

To reduce misclassification arising from transient elevation, we required that the retinal assessment and both biomarker measurements fall within a ±90-day window and that the patient be in a clinically stable outpatient state throughout. Patients were excluded if, within that window, they had a recorded episode of acute kidney injury (KDIGO criteria), acute coronary syndrome, decompensated heart failure, any hospitalisation, or a documented systemic infection.

## 3. Biomarker Definitions and Outcome Measures (Table 1)

### Statistical Analysis

Continuous variables are reported as mean ± SD (normally distributed) or median [IQR] (non-normal), assessed by the Kolmogorov–Smirnov test. Group comparisons used the independent-samples *t*-test or Mann–Whitney U test for continuous variables, and chi-square or Fisher’s exact test for categorical variables. Binary logistic regression estimated crude and adjusted ORs with 95% CIs across three sequential models: Model 1 (unadjusted), Model 2 (age- and sex-adjusted), Model 3 (fully adjusted for all pre-specified covariates in Section 2.2). Cohen’s weighted kappa (κ) with 95% CI was used to quantify inter-ophthalmologist agreement. Dose–response trend across DR severity grades was assessed by the Cochran–Armitage test. ROC curve analysis with AUC and 95% CI (2000-iteration bootstrap) evaluated discriminative accuracy. Variance inflation factors (VIF) assessed multicollinearity. A single primary hypothesis was pre-specified (the composite outcome), and no correction was applied to that single primary test. All remaining analyses—the individual biomarker comparisons, the baseline comparisons in Table 2, and the severity-gradient analysis—are secondary and exploratory. For these we additionally report a Benjamini–Hochberg false-discovery-rate threshold at q = 0.05; associations that do not survive this threshold are described as hypothesis-generating rather than confirmatory. Model construction followed a pre-specified confounder list rather than data-driven selection. With 134 composite events, the fully adjusted model with 12 covariates corresponds to approximately 11 events per variable, at the accepted threshold of 10; a reduced model restricted to age, sex, HbA1c, diabetes duration, and eGFR is therefore also reported as a sensitivity analysis. Linearity in the logit for continuous covariates was assessed by the Box–Tidwell procedure. Because eGFR is mechanistically related to both UACR and NT-proBNP, we report the primary model with and without eGFR to allow the reader to judge potential overadjustment. Further pre-planned sensitivity analyses excluded patients with eGFR < 30 mL/min/1.73 m^2^, stratified by eGFR category (≥60 vs. 30–59), and modelled UACR and NT-proBNP as continuous (log-transformed) variables. All analyses were performed using SPSS v.27 (IBM Corp., Armonk, NY, USA) and R v.4.3 (R Foundation, Vienna, Austria). Two-tailed *p* < 0.05 was considered statistically significant. Reporting adheres to STROBE guidelines [17].

**Table 1:** Operational definitions of primary variables.

Variable	Threshold/Definition	Reference
Nephropathy	UACR ≥ 30 mg/g	[14,16]
Subclinical Cardiac Stress	NT-proBNP ≥ 125 pg/mL	[10]
Primary Composite Outcome	Both UACR ≥ 30 mg/g AND NT-proBNP ≥ 125 pg/mL simultaneously	—
DR-Positive (Exposure)	Ophthalmologist consensus grade ≥ 1 (any NPDR or PDR)	[3,4,5]
DR-Negative (Reference)	Ophthalmologist consensus grade 0 (no DR)	[3,4,5]

**Table 2:** Baseline characteristics by ophthalmologist-graded DR status.

Characteristic	DR-Positive (*n* = 187)	DR-Negative (*n* = 214)	*p*-Value
Age (years), mean ± SD	64.1 ± 9.7	55.2 ± 10.5	<0.001
Female sex, *n* (%)	82 (43.9%)	110 (51.4%)	0.13
BMI (kg/m^2^), mean ± SD	30.6 ± 4.4	30.4 ± 5.0	0.65
Diabetes duration (years), mean ± SD	14.8 ± 6.0	8.2 ± 4.2	<0.001
HbA1c (%), mean ± SD	9.1 ± 1.5	7.5 ± 1.1	<0.001
eGFR (mL/min/1.73 m^2^), mean ± SD	57.7 ± 18.0	75.1 ± 15.7	<0.001
Systolic BP (mmHg), mean ± SD	139.2 ± 14.4	129.9 ± 13.1	<0.001
Hypertension, *n* (%)	135 (72.2%)	95 (44.4%)	<0.001
RAS blocker use, *n* (%)	134 (71.7%)	88 (41.1%)	<0.001
Statin use, *n* (%)	114 (61.0%)	127 (59.3%)	0.74
SGLT-2 inhibitor use, *n* (%)	66 (35.3%)	72 (33.6%)	0.73
Insulin use, *n* (%)	122 (65.2%)	65 (30.4%)	<0.001
UACR (mg/g), median [IQR]	100 (52–216)	21 (12–39)	<0.001
NT-proBNP (pg/mL), median [IQR]	210 (116–385)	62 (31–106)	<0.001

RAS = renin–angiotensin system; SGLT-2 = sodium–glucose cotransporter-2; UACR = urine albumin-to-creatinine ratio; NT-proBNP = N-terminal pro-B-type natriuretic peptide. Continuous: *t*-test or Mann–Whitney U; categorical: chi-square or Fisher’s exact.

## 4. Results

### 4.1. Study Population and Ophthalmological Assessment

Of 524 T2DM patients initially evaluated, 401 met all eligibility criteria and comprised the final study cross-sectional (Figure 1). Of these, 187 (46.6%) were classified as DR-positive and 214 (53.4%) as DR-negative by ophthalmologist consensus grade. Among DR-positive patients, 59 (31.6%) had Mild NPDR, 68 (36.4%) Moderate NPDR, 36 (19.3%) Severe NPDR, and 24 (12.8%) PDR. Seven patients whose two recorded grades were discordant at the DR-positive/negative level were resolved through the adjudication procedure described in Section 2.3; no external arbitration was required.

Inter-physician agreement was high across all five DR severity categories (Cohen’s weighted κ = 0.88; 95% CI: 0.83–0.93; *p* < 0.001). Agreement was similarly high at the dichotomous DR-positive/negative level (κ = 0.91; 95% CI: 0.86–0.96). These values are consistent with published benchmarks for experienced ophthalmologist panel grading in diabetic eye disease [4,5], confirming the diagnostic reproducibility of the grading protocol.

### 4.2. Baseline Characteristics

Baseline characteristics are presented in Table 2. DR-positive patients were significantly older (64.1 ± 9.7 vs. 55.2 ± 10.5 years; *p* < 0.001), had longer diabetes duration (14.8 ± 6.0 vs. 8.2 ± 4.2 years; *p* < 0.001), and higher HbA1c levels (9.1 ± 1.5 vs. 7.5 ± 1.1%; *p* < 0.001). eGFR was significantly lower in the DR-positive group (57.7 ± 18.0 vs. 75.1 ± 15.7 mL/min/1.73 m^2^; *p* < 0.001). Hypertension prevalence (135 (72.2%) vs. 95 (44.4%); *p* < 0.001), RAS blocker use (134 (71.7%) vs. 88 (41.1%); *p* < 0.001), and insulin use (122 (65.2%) vs. 65 (30.4%); *p* < 0.001) were significantly more prevalent in the DR group. Sex, BMI, statin use, and SGLT-2 inhibitor use did not differ significantly. Median UACR (100 (52–216) vs. 21 (12–39) mg/g; *p* < 0.001) and NT-proBNP levels (210 (116–385) vs. 62 (31–106) pg/mL; *p* < 0.001) were markedly higher in DR-positive patients.

### 4.3. Primary Outcome: Concurrent Nephropathy and Cardiac Stress

The primary composite outcome (UACR ≥ 30 mg/g AND NT-proBNP ≥ 125 pg/mL) was present in 120 (64.2%) DR-positive patients vs. 14 (6.5%) DR-negative patients (crude OR: 25.59 (13.78–47.51); *p* < 0.001). After full multivariable adjustment (Model 3), DR remained independently associated with the composite outcome (adjusted OR: 21.49 (11.02–41.81); *p* < 0.001). The very wide confidence intervals reflect the small number of composite events among DR-negative patients (*n* = 14) and should be interpreted with corresponding caution. Results across all three regression models are shown in Table 3.

### 4.4. Secondary Outcomes: Individual Biomarker Analyses

**Figure 2:**
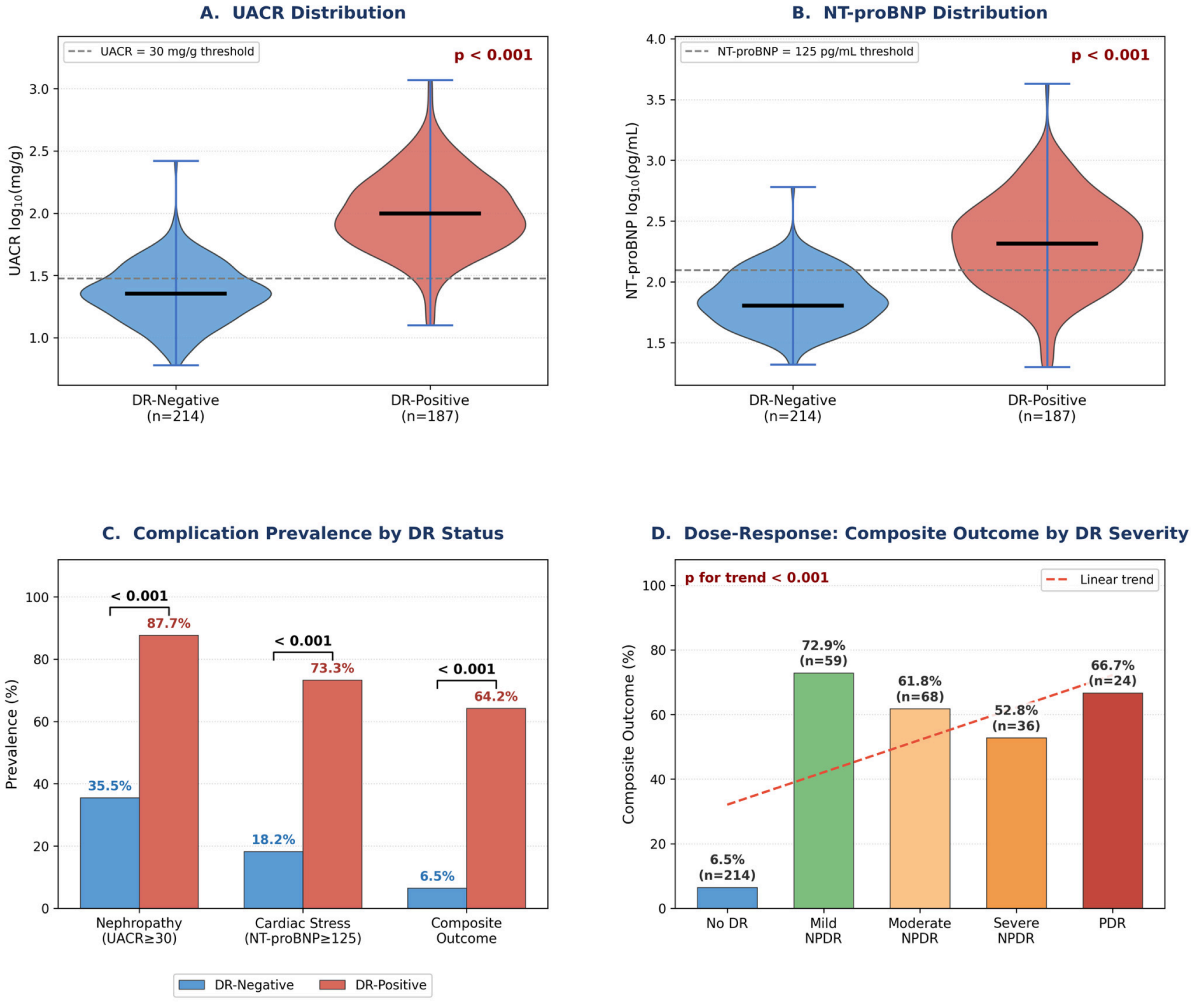
Biomarker profiles and complication burden by diabetic retinopathy status.

Nephropathy alone (UACR ≥ 30 mg/g) was present in 164 (87.7%) DR-positive vs. 76 (35.5%) DR-negative patients (OR: 12.95 (7.71–21.75); *p* < 0.001). Subclinical cardiac stress (NT-proBNP ≥ 125 pg/mL) was identified in 137 (73.3%) vs. 39 (18.2%) patients respectively (OR: 12.29 (7.65–19.76); *p* < 0.001). Both individual biomarker associations were highly significant and large in magnitude. Biomarker distributions and prevalence comparisons are illustrated in Figure 2.

### 4.5. Dose–Response Analysis by DR Severity

A statistically significant trend across ophthalmologist-graded DR severity categories was observed (Cochran–Armitage *p* for trend < 0.001); the pattern is, however, better described as a threshold effect at the transition from no DR to any DR than as a graded dose–response across severity strata. Composite outcome rates were: No DR 6.5%, Mild NPDR 72.9% (*n* = 59; composite *n* = 43), Moderate NPDR 61.8% (*n* = 68; composite *n* = 42), Severe NPDR 52.8% (*n* = 36; composite *n* = 19), and PDR 66.7% (*n* = 24; composite *n* = 16). The dominant discontinuity lies between no DR (6.5%) and any DR (≥52.8%). Beyond that threshold the gradient is not monotonic: composite outcome prevalence was numerically highest in Mild NPDR (72.9%) and lowest in Severe NPDR (52.8%), and the strata are small (Severe NPDR *n* = 36; PDR *n* = 24), so the differences between severity categories are compatible with sampling variation and no ordering among them should be inferred. We therefore interpret these data as showing that cardiorenal biomarker abnormality is already common at the earliest ophthalmoscopically detectable stage of retinopathy, and we make no claim of a progressive dose–response across DR grades.

### 4.6. ROC Analysis

**Figure 3:**
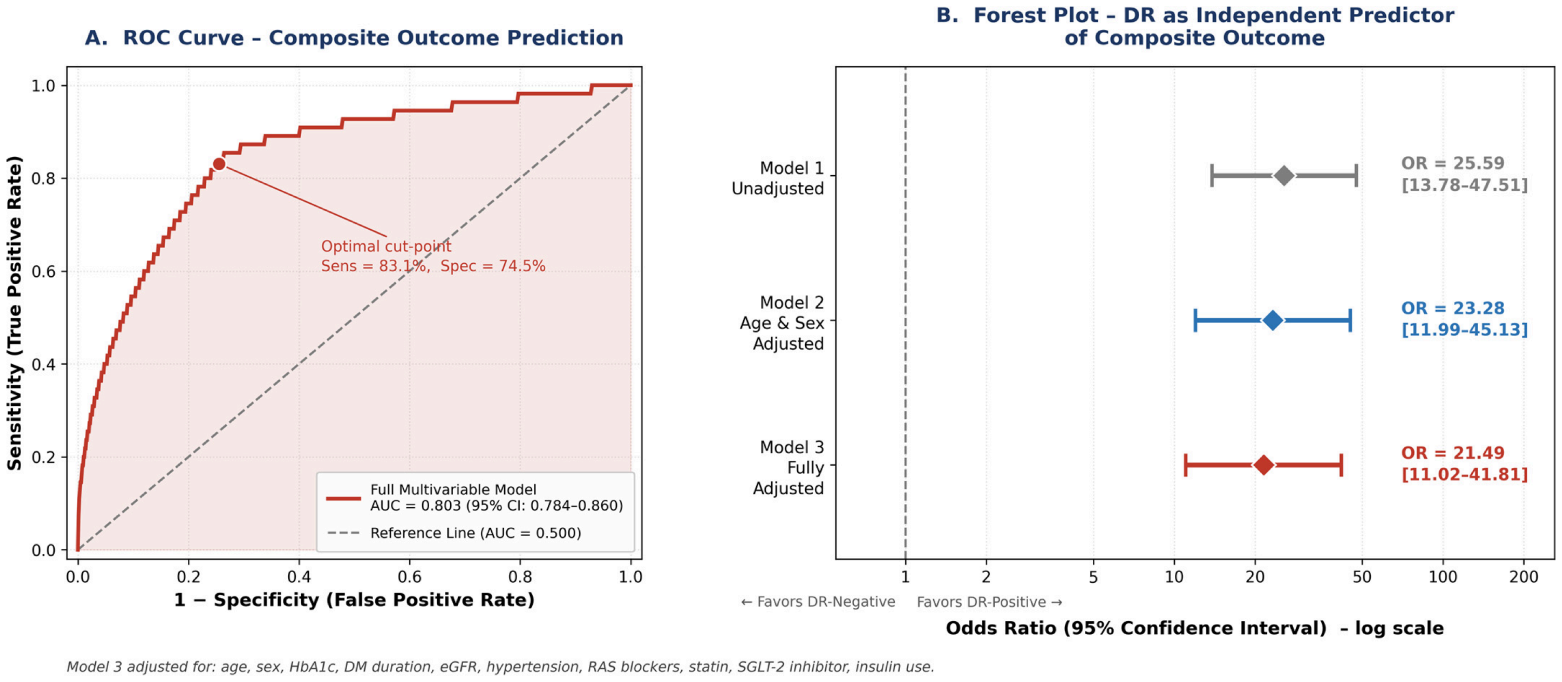
ROC curve and forest plot of adjusted odds ratios.

The full multivariable model incorporating ophthalmologist-graded DR status alongside age, HbA1c, diabetes duration, eGFR, and hypertension yielded an AUC of 0.837 (95% CI: 0.784–0.860) for the composite outcome. The Youden-optimal cut-point corresponded to a sensitivity of 83.1% and specificity of 74.5%. Because this AUC was obtained in the same sample used to fit the model, it reflects apparent rather than validated discrimination and is likely optimistic; no internal validation by bootstrap optimism correction or cross-validation was performed. The ROC curve and forest plot are presented in Figure 3.

## 5. Discussion

This retrospective cross-sectional study demonstrates that diabetic retinopathy—confirmed through expert ophthalmological examination by two independent board-certified ophthalmologists each with ≥ 10 years of dedicated experience, using ETDRS-based dilated fundus assessment—is strongly and independently associated with the concurrent presence of nephropathy (UACR ≥ 30 mg/g) and subclinical cardiac stress (NT-proBNP ≥ 125 pg/mL) in patients with T2DM. The crude OR of 25.59 was only modestly attenuated to 21.49 after full multivariable adjustment. Because exposure and outcome were ascertained at essentially the same point in time, this reflects co-occurrence rather than prediction, and no temporal sequence between retinal, renal and myocardial involvement can be inferred from these data. The high inter-physician agreement (weighted κ = 0.88) is consistent with published benchmarks for experienced ophthalmologist grading [4,5] and supports the reproducibility of the exposure classification.

The high prevalence of nephropathy (87.7%) among DR-positive patients is consistent with extensive prior literature supporting a shared pathophysiological basis for retinal and glomerular microvascular injury in diabetes [6,7]. Longitudinal data have established that DR severity is a robust independent predictor of CKD progression: NPDR confers approximately a three-fold and PDR a 16-fold higher risk of CKD progression compared to patients without DR [8]. These findings have been replicated in ethnically diverse cohorts, including a retrospective study of Latino T2DM patients at the Joslin Diabetes Centre, where moderate-to-severe NPDR and PDR were independently associated with significant eGFR decline [9].

The association between DR and nephropathy is, as the above literature shows, already well established, and we do not present it as novel. The more distinctive element of the present analysis is the simultaneous assessment of albuminuria and NT-proBNP against the same ophthalmologist-adjudicated retinal grade, and in particular the observation that the two abnormalities co-occur in nearly two-thirds of DR-positive patients (73.3% vs. 18.2% for NT-proBNP alone; OR: 12.29). NT-proBNP is a guideline-endorsed marker of myocardial wall stress and subclinical ventricular dysfunction, elevated in the setting of diabetic cardiomyopathy long before symptomatic heart failure develops [10,11]. A landmark hospital-based cohort demonstrated that NT-proBNP in the highest tertile was independently associated with an OR of 13.78 for diabetic retinopathy and 6.23 for nephropathy, establishing NT-proBNP as a global biomarker of diabetic vascular organ damage [11]. NT-proBNP has additionally been shown to correlate with pulse wave velocity and ankle-brachial index in T2DM patients, reflecting its utility as a marker of subclinical atherosclerosis [12]. A prospective European case–cohort study confirmed that higher NT-proBNP is independently associated with both microvascular (HR: 1.20) and macrovascular (HR: 1.37) complications among individuals who develop T2DM [13]. These findings are compatible with the concept that the retinal fundus reflects concurrent systemic cardiometabolic burden. An important caveat applies, however: NT-proBNP rises with declining renal clearance as well as with myocardial wall stress, and our composite outcome pairs it with a renal marker in a cohort whose DR-positive stratum had substantially lower eGFR (57.7 vs. 75.1 mL/min/1.73 m^2^). Part of the observed NT-proBNP elevation is therefore likely renal rather than cardiac in origin, and the two components of the composite are not independent of one another. Without echocardiography we cannot separate these contributions, and an elevated NT-proBNP in an asymptomatic patient should be read as an indication for further cardiac evaluation, not as a diagnosis of heart failure.

The step change in composite outcome prevalence between patients without DR (6.5%) and those with any DR (52.8–72.9%) aligns with the established principle that microvascular damage across organ systems tends to occur in parallel [1,2,6]. The magnitude of that step, rather than any ordering within the DR-positive strata, is the observation we consider interpretable; composite outcome prevalence was already 72.9% at the Mild NPDR stage, which suggests that cardiorenal biomarker abnormality is not confined to advanced retinopathy. A multicentric Italian cross-sectional study found that over 28% of asymptomatic T2DM patients with hypertension already had NT-proBNP levels consistent with subclinical cardiac stress [18], while microvascular endothelial dysfunction has been associated with higher risk of heart failure with preserved ejection fraction in women with T2DM [19], and NT-proBNP-based screening strategies have been shown to efficiently identify high-risk diabetic patients expected to benefit most from preventive cardioprotective therapies [20].

Any clinical implication of these findings must be stated conditionally. Fundus examination is already a standard component of diabetic care guidelines [16], and the present data raise the possibility that retinal grading carries information about concurrent cardiorenal status. Whether acting on that information improves outcomes is a question this study cannot answer, and we explicitly do not recommend any change to current screening practice on the basis of a single-centre cross-sectional analysis. Prospective work would need to establish, at minimum, that retinal grading adds discrimination beyond routinely available variables such as age, diabetes duration, HbA1c and eGFR, and that biomarker-triggered intensification of therapy—for example with nephroprotective agents such as finerenone [21,22] or cardioprotective SGLT-2 inhibitors [23,24], both of which have demonstrated renal and cardiovascular benefit in high-risk T2DM [21,22,23,24]—yields better outcomes than usual care in this specific population.

## 6. Limitations

Several limitations must be acknowledged, and a number of them constrain the interpretation of our findings substantially.

**Design and inference.** The design is retrospective and cross-sectional. Exposure and outcome were ascertained at essentially the same time, so the study can demonstrate association but cannot establish temporal sequence, prediction, causality, or clinical utility. Concurrent retinopathy, albuminuria and elevated NT-proBNP may simply reflect shared cumulative hyperglycaemic exposure and shared duration of disease rather than any causal chain between them.

**Non-independence of the composite outcome.** This is, in our view, the most important limitation. NT-proBNP is not a pure marker of myocardial wall stress: it is cleared renally and rises as eGFR falls, and it is also lowered by obesity, a relevant consideration in a cohort with a mean BMI above 30 kg/m^2^ in both groups. Our composite outcome therefore combines a renal marker (UACR) with a second marker that is itself partly renal. Because the DR-positive group had markedly lower eGFR, an unknown proportion of the association we report between DR and the composite outcome is attributable to renal function acting on both components rather than to independent cardiac involvement. Adjustment for eGFR mitigates but cannot eliminate this, and adjusting for a variable that lies on the causal path between the exposure and both outcome components risks overadjustment in the opposite direction. The label “subclinical cardiac stress” should accordingly be read as shorthand for “NT-proBNP above the ESC rule-out threshold”, not as a validated cardiac phenotype.

**Absence of cardiac imaging.** No echocardiographic data were available. An elevated NT-proBNP in an asymptomatic patient is not a diagnosis of heart failure; it is an indication for further diagnostic evaluation. Without structural or functional cardiac assessment we cannot confirm that the elevated values reflect genuine subclinical ventricular dysfunction, and we cannot exclude alternative causes such as unrecognised valvular disease or pulmonary pathology.

**Indication-based selection for NT-proBNP.** NT-proBNP is not routinely measured in all patients with type 2 diabetes and has no guideline-mandated role in uncomplicated diabetes follow-up. It was ordered by treating clinicians on clinical suspicion, which means the analysed cohort is enriched for patients in whom cardiorenal involvement was already suspected. The prevalence estimates reported here are therefore not generalisable to unselected T2DM populations, and the magnitude of the DR–NT-proBNP association may be inflated relative to what would be observed if the biomarker were measured in everyone.

**Selection imposed by the dual-grading requirement.** Eligibility required that two independent retinal gradings already exist in the record. Patients graded by a single ophthalmologist during the same period were not eligible, and it is possible that dual grading was more likely in patients with more complex or more advanced eye disease. This may bias the DR-positive stratum toward greater severity. Exclusion of patients with media opacity precluding fundus visualisation acts in the same direction, since cataract severity correlates with diabetes duration.

**Incomplete medication data.** Table 2 reports only SGLT-2 inhibitor, insulin, RAS blocker and statin use. We did not extract the use of metformin, sulfonylureas, thiazolidinediones, DPP-4 inhibitors, GLP-1 receptor agonists, or dual GIP/GLP-1 agonists. This omission matters for two reasons. First, incretin-based agents are directly relevant to the retina: GLP-1 receptors are expressed in retinal tissue, and rapid glycaemic improvement and rapid weight loss are both recognised precipitants of early worsening of diabetic retinopathy [7]. An unmeasured imbalance in incretin use between groups could therefore confound the DR classification itself. Second, GLP-1 receptor agonists and SGLT-2 inhibitors both reduce albuminuria and modify natriuretic peptide levels, so they act directly on both components of our composite outcome.

**Absent lipid data.** Lipid profile was not extracted, although statin use was recorded. Dyslipidaemia is associated with both diabetic retinopathy—particularly with hard exudates and diabetic macular oedema—and with albuminuria, and its omission leaves open a further source of residual confounding.

**Single UACR measurement and the biomarker window.** UACR was based on a single spot measurement. Guidelines require confirmation across two to three samples over three to six months before albuminuria is considered established [14,16], and intra-individual biological variability in spot UACR is substantial, so some patients will have been misclassified. The ±90-day window between retinal assessment and biomarker sampling is clinically wide for both analytes: NT-proBNP responds to volume status within days, and albuminuria fluctuates with glycaemic control, blood pressure, intercurrent illness and medication changes. Non-differential misclassification of this kind would ordinarily bias toward the null, but we cannot exclude differential misclassification if DR-positive patients were sampled during less stable clinical periods.

**Statistical limitations.** The fully adjusted model contains 12 covariates for 134 composite events, which is close to the conventional lower bound for events per variable and raises the possibility of model overfitting and unstable estimates; the very wide confidence intervals around the primary odds ratio are consistent with this. The multiple secondary comparisons were not corrected in the originally submitted analysis, and although the primary association is of a magnitude that would survive any plausible correction, the secondary and subgroup findings should be regarded as exploratory. The reported AUC is an apparent, in-sample estimate and has not been internally validated, so true discrimination in new patients is likely to be lower.

**Non-monotonic severity gradient.** Composite outcome prevalence did not increase monotonically across DR severity strata, and the higher-severity strata are small. We have therefore refrained from describing a progressive dose–response and interpret the data only as showing a threshold difference between the absence and presence of retinopathy.

**Generalisability.** This is a single-centre study conducted in one region of Türkiye. The findings may not generalise to other ethnic groups, healthcare systems, or settings with different thresholds for ordering NT-proBNP.

## 7. Strengths

Set against these limitations, the study has some methodological strengths. The exposure was classified by two independent board-certified ophthalmologists, each with at least 10 years of dedicated diabetic eye disease experience, using standardised ETDRS-based dilated fundus examination, with documented and high inter-physician reproducibility (κ = 0.88) consistent with published expert benchmarks [4,5]—a level of exposure ascertainment uncommon in retrospective cardiorenal studies, which more often rely on coded diagnoses or single-grader photographic screening. Two validated, guideline-endorsed biomarkers addressing distinct organ systems were evaluated in the same patients at the same time [10,14,16], multivariable adjustment included pharmacological treatment, and the entirely routine-care origin of the data means the associations described reflect an unselected real-world clinical workflow rather than a research protocol.

## 8. Conclusions

In this single-centre retrospective cross-sectional study, ophthalmologist-graded diabetic retinopathy was strongly associated with the concurrent presence of albuminuria and elevated NT-proBNP in adults with type 2 diabetes. The design permits no inference about temporal sequence, causality or clinical utility, and part of the observed association is likely attributable to shared renal determinants of both markers. Prospective multicentre studies with cardiac imaging, confirmed albuminuria and complete medication data are needed to determine whether retinal grading adds incremental value for cardiorenal risk stratification beyond standard clinical variables.

## Figures and Tables

**Table 3:** Logistic regression: ophthalmologist-confirmed DR as independent predictor of composite outcome.

Regression Model	OR (95% CI)	*p*-Value	Covariates
Model 1—Unadjusted	25.59 (13.78–47.51)	<0.001	None
Model 2—Age- & Sex-Adjusted	23.28 (11.99–45.13)	<0.001	Age, sex
Model 3—Fully Adjusted *	21.49 (11.02–41.81)	<0.001	Age, sex, HbA1c, DM duration, eGFR, hypertension, RAS blockers, statin, SGLT-2, insulin, BMI, SBP

* All VIF < 2.8. OR = odds ratio; CI = confidence interval.

## Data Availability

Data are available upon request from the authors.
